# Photosystem II Responses at the Whole-Potato-Leaf Level After Colorado Potato Beetle Feeding

**DOI:** 10.3390/plants15081159

**Published:** 2026-04-09

**Authors:** Ilektra Sperdouli, Stefanos S. Andreadis, Julietta Moustaka, Eleni I. Koutsogeorgiou, Emmanuel Panteris, Michael Moustakas

**Affiliations:** 1 Institute of Plant Breeding and Genetic Resources, Hellenic Agricultural Organization-Dimitra, 57001 Thessaloniki, Greece; esperdouli@elgo.gr (I.S.); sandreadis@elgo.gr (S.S.A.); ekoutsogeorgiou@gmail.com (E.I.K.); 2 Department of Food Science, Aarhus University, 8000 Aarhus, Denmark; julietta_moustaka@food.au.dk; 3 Department of Botany, School of Biology, Aristotle University of Thessaloniki, 54124 Thessaloniki, Greece; epanter@bio.auth.gr

**Keywords:** chlorophyll fluorescence, ROS, light-energy use efficiency, singlet oxygen, non-photochemical quenching, insect herbivory, reaction centers, photoinhibition, hydrogen peroxide, photosynthetic heterogeneity

## Abstract

The damage caused by herbivores is generally measured as the amount of leaf tissue consumed, without accounting for the fate of the leftover tissue. As a result, the plant defense mechanisms that promote resistance to herbivore feeding by photosynthetically acclimating the rest of the plant to the feeding spot leaf area have not been well exploited. Plant-insect interactions are now becoming better defined with the development of visualization methods that permit spatial whole-leaf assessment of photosynthetic efficiency after herbivore attack. The purpose of our study was to evaluate the spatial heterogeneity of photosystem II (PSII) function at the whole-leaf level before and after herbivory by the Colorado potato beetles. Twenty minutes after Colorado potato beetle (*Leptinotarsa decemlineata*) feeding, the maximum efficiency of PSII photochemistry (F*v*/F*m*) decreased significantly, suggesting photoinhibition due to reduced efficiency of the oxygen-evolving complex (OEC). The decreased quantum yield of PSII photochemistry (Φ*_PSII_*) after feeding, at the neighboring area of the feeding spot and at the rest of the leaf area, was attributed to the reduced efficiency of the open PSII reaction centers (F*v′*/F*m′*), since there was no change in the fraction of open PSII reaction centers (q*p*). Nevertheless, plant defense elicitation was activated by the photoprotective mechanism of non-photochemical quenching (NPQ) that reduced the singlet oxygen (^1^O_2_) formation in potato plants in the neighboring area of the feeding spot and at the rest of the leaf area. In addition, the increased production of hydrogen peroxide (H_2_O_2_) triggered by this increase suggests that it acted as a signaling molecule in the biotic stress defense response.

## 1. Introduction

Herbivorous insects pose a major agricultural challenge, reducing annual crop yields and resulting in considerable losses, currently estimated at approximately 38% [1]. Traditional approaches to estimating productivity losses due to herbivory in agricultural systems often overlook the effects on photosynthesis in the remaining undamaged leaf tissue [2,3,4]. High-resolution analysis of photosynthetic function is therefore essential for elucidating how intact areas are physiologically influenced [3]. Chlorophyll fluorescence analysis has emerged as a basic methodological tool for probing the functional integrity of the photosynthetic apparatus and for quantifying plant tolerance to diverse biotic and abiotic stressors [4,5,6,7,8,9]. Since stress conditions perturb the utilization efficiency of absorbed light energy, chlorophyll fluorescence provides a sensitive, noninvasive, cost-effective, and highly accurate diagnostic tool for stress-induced impairments in photosynthetic efficiency [3,6,7,10,11,12,13,14]. However, the spatial heterogeneity of photosynthetic activity across the leaf lamina limits the usefulness of standard point-based chlorophyll fluorescence measurements [8,15,16,17,18,19]. The development of chlorophyll fluorescence imaging apparatus has substantially overcome this limitation by enabling spatially resolved quantification of photosynthetic heterogeneity at the whole-leaf scale [15,17,19,20].

Insect herbivory, like other biotic stresses, is known to alter photosynthetic activity, most commonly reducing it, although compensatory enhancements can also occur [3,7,12,13,21,22]. Because the light-dependent reactions generate the reducing power and chemical energy necessary for synthesizing a broad suite of defense-related metabolites, they constitute a critical element of the plant’s integrated response to herbivore attack [23]. Photosystem II (PSII), a multiunit thylakoid membrane complex responsible for photochemical charge separation, water oxidation, and the generation of atmospheric oxygen, is indispensable for sustaining aerobic life on Earth [5,24,25]. PSII is also widely recognized as one of the most stress-sensitive components of the photosynthetic apparatus, exhibiting pronounced susceptibility to both biotic and abiotic perturbations [15,26,27].

Insect herbivory triggers plant responses involving photosynthesis, reactive oxygen species (ROS), and hormonal signaling, which are interconnected through a complex crosstalk [28,29]. Primarily, herbivory affects photosynthesis and initiates defense mechanisms, such as ROS production and the release of defense hormones, including jasmonic acid (JA) and salicylic acid (SA) [3,28]. ROS, such as singlet oxygen (^1^O_2_), hydrogen peroxide (H_2_O_2_), and superoxide anion radical (O_2_^•−^), are continuously produced in the light reactions of photosynthesis at basal levels, and are kept under optimal growth conditions by the antioxidative enzymatic and non-enzymatic systems in homeostasis [11,29,30,31,32]. However, under environmental stress conditions, the equilibrium between ROS generation and removal is disrupted [32,33,34,35,36].

ROS, acting as key signaling molecules, combine hormonal and photosynthetic pathways to coordinate a defense response [28]. ROS generation in photosynthesis plays a fundamental role as retrograde signaling molecules, activating the plant’s defense responses to environmental stressors, contributing to the “oxidation-reduction” balance, regulating a variety of physiological functions, and activating a plethora of acclimation responses [29,30,37,38,39,40,41]. Plants not only respond to insect feeding damage but also respond to insect egg positioning at the earliest stage of insect attack, allowing the plant to prepare its defense even before the damaging feeding stages of the insect life cycle have begun [42,43].

Potato (*Solanum tuberosum* L.) is one of the most important world food crops, ranking third after rice and wheat [44,45], and is significant because of its starch-rich tubers, which are a principal source of the daily diet for several people [46]. Potatoes are vulnerable to many types of biotic stress, with defoliating insects posing a major threat that can diminish both yield and tuber quality [12]. Among these insect pests, the Colorado potato beetle (*Leptinotarsa decemlineata* Say, Coleoptera: Chrysomelidae) is particularly notable for the extensive damage it causes and its remarkable resistance to insecticides [45]. The Colorado potato beetle is currently considered to be the principal insect defoliator of potatoes. Both adults and larvae consume whole leaves without differentiating between leaf tissues. Climate change may significantly affect the behavior and distribution of Colorado potato beetles, allowing them to spread into regions where they were not previously found, thereby increasing their harmfulness [47,48]. Yet, due to global climate change, increased drought stress affects plant health, resulting in fewer resistant plants against pest attacks [4].

Here, we studied the effects of short-time-restricted feeding by the most damaging phytophagous pest of potato on light energy use efficiency and ROS generation. We aimed to assess whether the photosynthetic mechanism could respond to Colorado potato beetle feeding and prevent further damage to the remaining leaf area. We investigated whether the leaf parts not damaged by herbivory (the neighboring area to the feeding spot and the rest of the leaf area) were negatively affected after herbivore feeding, or whether the photoprotective mechanism triggered defense elicitation to prevent further damage.

## 2. Results

### 2.1. Spatial Heterogeneity of PSII Function Before and After Feeding

**Figure 1:**
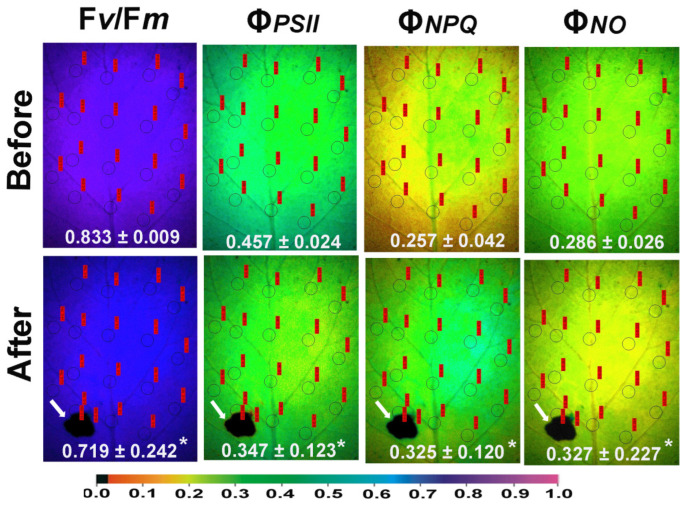
Representative images of the maximum efficiency of PSII photochemistry (F*v*/F*m*), the effective quantum yield of PSII photochemistry (Φ*_PSII_*), the quantum yield of regulated non-photochemical energy loss in PSII (Φ*_NPQ_*), and the quantum yield of non-regulated energy loss in PSII (Φ*_NO_*) before and 20 min after Colorado potato beetle feeding. The white arrows show the feeding spot area in potato leaflets. The areas of interest (AOIs) measured at the leaf surface are shown as circles, while for each parameter, the overall mean value from n = 3–4 leaves (±SD) is given in white. White arrows point to feeding areas. Two areas of interest (AOIs), shown as circles with their associated measurements labeled in red, were added at the feeding spot. An asterisk denotes statistical significance difference between before and after feeding. The color code on the bottom of the images shows pixel values ranging from 0.0 to 1.0.

The potato leaflets show photosynthetic heterogeneity both before and after herbivore feeding. In the representative color-coded images of the chlorophyll fluorescence parameters, an obvious spatial heterogeneity between the left and right leaf sides was recognized, with higher values of the maximum efficiency of PSII photochemistry (F*v*/F*m*), and a higher amount of absorbed light energy directed to photochemistry (Φ*_PSII_*), occurring in the left leaf side (Figure 1). The higher values of the quantum yield of regulated non-photochemical energy loss in PSII (Φ*_NPQ_*) occurred at the right side, while the higher values of the quantum yield of non-regulated energy loss in PSII (Φ*_NO_*) occurred at the left leaf side (Figure 1). This spatial heterogeneity between the left and right leaf sides was also retained after herbivore feeding. Twenty min of herbivore feeding resulted in decreased whole leaf F*v*/F*m* and Φ*_PSII_* values and increased whole leaf Φ*_NPQ_* and Φ*_NO_* values (Figure 1). The white arrows in Figure 1 show the feeding spot area, wherein the two areas of interest (AOIs) and their associated values for the maximum efficiency of PSII photochemistry (F*v*/F*m*), the effective quantum yield of PSII photochemistry (Φ*_PSII_*), and the quantum yield of regulated non-photochemical energy loss in PSII (Φ*_NPQ_*) were all equal to 0.00. However, the associated values for the quantum yield of non-regulated energy loss in PSII (Φ*_NO_*) were 1.00 (Figure 1).

### 2.2. Impact of Feeding on the Efficiency of the Oxygen-Evolving Complex and the Maximum Efficiency of Photosystem II Photochemistry

**Figure 2:**
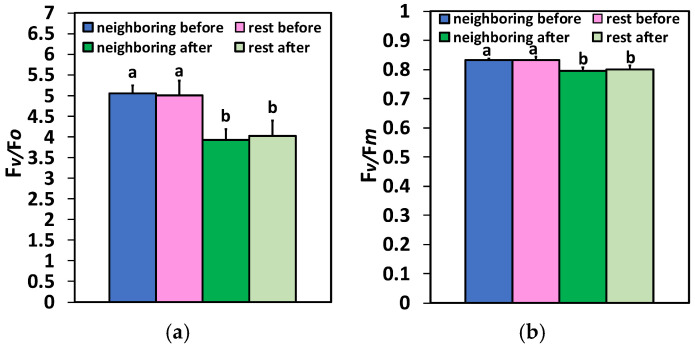
Changes in the efficiency of the oxygen-evolving complex (F*v*/F*o*) (**a**) and the maximum efficiency of PSII photochemistry (F*v*/F*m*) (**b**) in the two different zones (the neighboring area at the feeding spot and the rest of the leaf area) before and 20 min after Colorado potato beetle feeding. Bars display standard deviations (SD). Significant differences are shown by different lower-case letters (*p* < 0.05).

The efficiency of the oxygen-evolving complex (OEC) as evaluated in the two different zones (the neighboring area at the feeding spot and the rest of the leaf area) by the ratio F*v*/F*o*, was significantly lower 20 min after feeding compared to before (Figure 2a). No difference between the two zones, before or after feeding, was observed. Similar results were observed for the maximum efficiency of PSII photochemistry (F*v*/F*m*) in the two areas before and after feeding, with lower F*v*/F*m* values detected after feeding (Figure 2b).

### 2.3. Impact of Feeding on the Light Energy Use Efficiency

The absorbed light energy is allocated either to photochemistry (Φ*_PSII_*), dissipated as heat (ΦNPQ), or lost in a nonregulatory way (ΦNO), with all of them being equal to 1 [49].

**Figure 3:**
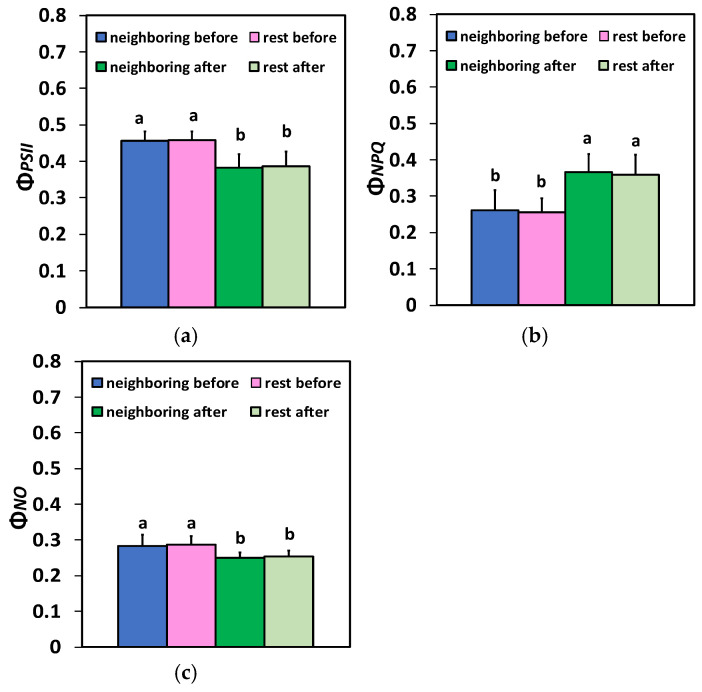
Changes in Φ*_PSII_* (**a**), Φ*_NPQ_* (**b**), and Φ*_NO_* (**c**) in the two different zones (the neighboring area at the feeding spot and the rest of the leaf area) before and 20 min after Colorado potato beetle feeding. Bars display standard deviations (SD). Significant differences are shown by different lower-case letters (*p* < 0.05).

The quantum yield for photochemistry (Φ*_PSII_*) for the neighboring area at the feeding spot and for the rest of the leaf was significantly lower 20 min after feeding compared to before feeding (Figure 3a). In contrast to Φ*_PSII_*, the regulated non-photochemical energy loss in PSII (Φ*_NPQ_*) was significantly higher 20 min after feeding than before, in both areas (Figure 3b). Also, no difference was observed between the neighboring area at the feeding spot and the rest of the leaf in Φ*_NPQ_* values, both before and after feeding (Figure 3a).

The yield of non-regulated energy loss in PSII (Φ*_NO_*) was significantly lower 20 min after feeding compared to before feeding, at the neighboring area to the feeding spot and at the rest of the leaf (Figure 3c). This decrease contrasted with the observed increase in Φ*_NO_* values after feeding compared to before, evaluated across the whole leaf area (Figure 1). This was due to the high Φ*_NO_* values (1.00) detected at the feeding spot, where the two areas of interest (AOIs) shown as circles in Figure 1 were added at the feeding spot.

### 2.4. Impact of Feeding on the Fraction of Open PSII Reaction Centers, Their Efficiency, the Electron Transport Rate, and the Photoprotective Mechanism

**Figure 4:**
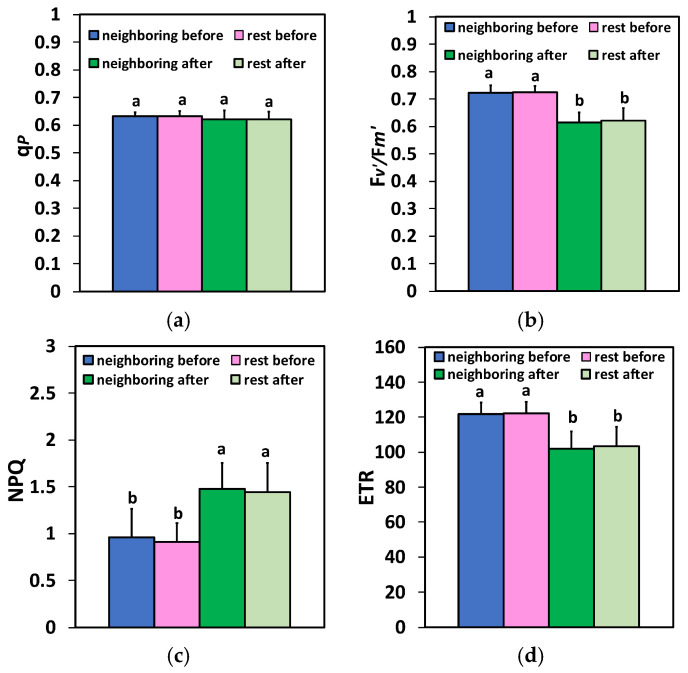
Changes in the portion of open PSII reaction centers (RCs) (q*p*) that reveal the redox state of quinone A (Q_A_) (**a**), the efficiency of the open PSII RCs (F*v′*/F*m′*) (**b**), the photoprotective mechanism of non-photochemical quenching (NPQ) (**c**), and the electron transport rate (ETR) (**d**) in the two different zones (the neighboring area at the feeding spot and the rest of the leaf area) before and 20 min after Colorado potato beetle feeding on potato leaves. The bars display standard deviations (SD). Significant differences are shown by different lower-case letters (*p* < 0.05).

The fraction of open PSII reaction centers (RCs) (q*p*), representing the redox state of quinone A (Q_A_), after 20 min of feeding, remained the same as before feeding (Figure 4a). No differences were observed between the neighboring area at the feeding spot and the rest of the leaf in q*p* values, both before and after feeding (Figure 4a). A reduced efficiency of the open PSII reaction centers (RCs) (F*v′*/F*m′*) at the neighboring area of feeding and at the rest of the leaf was observed 20 min after feeding, compared with before feeding (Figure 4b). No differences were observed between the neighboring area at the feeding spot and the rest of the leaf in the efficiency of the open PSII RCs (F*v′*/F*m′*), both before and after feeding (Figure 4b).

The photoprotective mechanism of non-photochemical quenching (NPQ) increased significantly 20 min after feeding, compared with before, in both areas (Figure 4c). However, no difference was observed between the neighboring area at the feeding spot and the rest of the leaf in NPQ values, both before and after feeding (Figure 4c). The electron transport rate (ETR) reduced significantly at the neighboring area of the feeding spot and at the rest of the leaf 20 min after the feeding compared to before feeding (Figure 4d).

### 2.5. Correlation of the Maximum Efficiency of PSII with the Efficiency of the Oxygen-Evolving Complex

**Figure 5:**
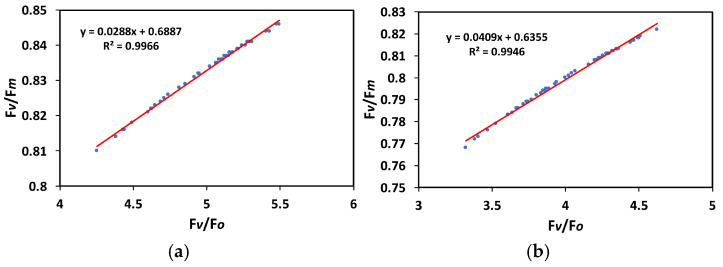
The relationship of the maximum efficiency of PSII photochemistry (F*v*/F*m*) with the efficiency of the oxygen-evolving complex (F*v*/F*o*) before (**a**) and after 20 min of Colorado potato beetle feeding on potato leaves (**b**). The blue dots represent the corresponding measurements of the variables, while the red line is the regression line that shows the relationship between the two variables.

The maximum efficiency of photosystem II photochemistry (F*v*/F*m*) and the efficiency of the oxygen-evolving complex (F*v*/F*o*) were positive and significantly correlated as shown in a regression analysis for both before feeding (R^2^ = 0.9966, *p* < 0.01) (Figure 5a), and after 20 min of Colorado potato beetle feeding on potato leaves (R^2^ = 0.9946, *p* < 0.01) (Figure 5b).

### 2.6. Hydrogen Peroxide Imaging Before and After Herbivore Feeding

**Figure 6:**
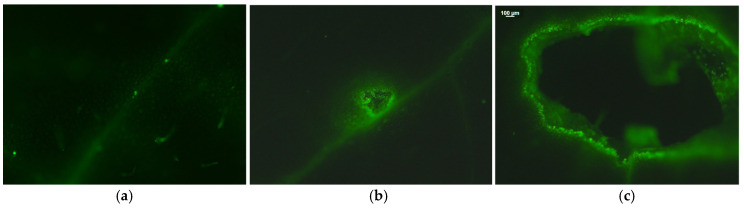
H_2_O_2_ production in potato leaves before (**a**) and after 20 min of Colorado potato beetle feeding (**b**,**c**). The light green color denotes H_2_O_2_ generation. The magnification in (**c**) is the feeding spot area.

Hydrogen peroxide production in potato leaves was localized mainly in the leaf veins, as evidenced by green fluorescence (Figure 6). After 20 min of Colorado potato beetle feeding, H_2_O_2_ generation was localized at the feeding spot (Figure 6b,c) and at the leaf veins (Figure 6b), where it was slightly increased compared to control leaves (Figure 6a). H_2_O_2_ was not detected at the neighboring of the feeding spot or at the rest of the leaf area (Figure 6b,c).

## 3. Discussion

Plant-insect interactions are now becoming better defined with the development of visualization methods that permit spatial whole-leaf assessment of the distribution of absorbed light energy before and after herbivore attack [3,13,15]. Under many biotic or abiotic stress factors, the absorbed light energy in photosynthesis exceeds the capacity of de-excitation processes, leading to photoinhibition and primarily damaging PSII via the formation of ROS [29,30,33,34,35,36,50]. Twenty minutes after Colorado potato beetle feeding, the maximum efficiency of PSII photochemistry (F*v*/F*m*) decreased significantly, suggesting possible photoinhibition. Photoinhibition appears either at the acceptor side through the excited triplet state of chlorophyll (^3^Chl*), resulting in singlet oxygen (^1^O_2_) formation, or at the donor side through inactivation of the OEC [51,52,53,54,55,56,57,58]. The OEC catalyzes water oxidation, located on the electron donor side of PSII, and contains the so-called Mn_4_CaO_5_ cluster [59,60,61]. Consequently, photoinhibition can emerge via a dual mechanism, with PSII as the primary target [53,55,62]. The observed photoinhibition in potato leaves 20 min after Colorado potato beetle feeding was the result of an impairment of the OEC function, as estimated by the decreased F*v*/F*o* ratio [63,64,65,66,67,68,69]. It is well known that reduced OEC efficiency also implies a lower F*v*/F*m* ratio [70,71]. In agreement with this, a significant positive correlation between the maximum efficiency of PSII photochemistry (F*v*/F*m*) and the function of OEC was revealed by regression analysis, both before (Figure 5a) and after feeding by the Colorado potato beetle (Figure 5b). Photoinhibition, which is associated with reduced OEC efficiency [72,73,74,75], is often associated with crop production [76].

However, since advanced analysis beyond simple F*v*/F*m* is required for accurate physiological assessment [77], we examined the light-use efficiency of potato plants before and after 20 min of Colorado potato beetle feeding. The observed photoinhibition in potatoes after the feeding, as observed by the reductions in F*v*/F*m*, was coupled with decreases in the effective quantum yield of PSII photochemistry (Φ*_PSII_*) (Figure 1). The decreased photochemical efficiency (Φ*_PSII_*), at the neighboring area to the feeding spot and at the rest of the leaf area after Colorado potato beetle feeding (Figure 3a), was overcompensated by the amplified regulated non-photochemical energy loss in PSII (Φ*_NPQ_*) (Figure 3b), which developed a drop in non-regulated loss (Φ*_NO_*) (Figure 3c). This decreased Φ*_NO_* is correlated with reduced quantity of singlet oxygen (^1^O_2_) generation [11,78,79,80]. ^1^O_2_ is created through the interaction of molecular O_2_ with the excited ^3^Chl* and is considered highly reactive and damaging [30,81,82,83,84]. Thus, the reduced ^1^O_2_ generation at the neighboring area of the feeding spot and at the rest of the leaf area, after 20 min of Colorado potato beetle feeding, confirms that the observed photoinhibition was associated with a “donor-side photoinhibition” through a reduced OEC efficiency. Otherwise, an increased ^1^O_2_ production illuminates the so-called ‘‘acceptor-side photoinhibition’’ of PSII [85,86,87,88,89]. However, a 20 min feeding by the pinworm *Tuta absoluta* on potato leaves did not have any effect on the OEC or F*v*/F*m* at the whole-leaf level [12]. Inhibition of photosynthesis due to insect herbivory was frequently reported [12,21,90,91,92], and in this context, ROS play a crucial role [12,28].

Plants can respond to disturbances in their homeostasis, triggered by biotic stress factors, by displaying adaptive responses that often result in compensatory responses [4,25,32]. Understanding these molecular mechanisms that initiate adaptive responses in plants is significant for enhancing crop productivity [4]. Thus, plants have developed several photoprotective mechanisms to counteract ROS production and hence escape PSII damage [29,32,93,94]. The dissipation of excess absorbed light energy as heat by the mechanism of non-photochemical quenching (NPQ) [31,81,95,96] protects PSII from the formation of ROS, which are considered detrimental to plant cells [31,81,97,98,99]. Twenty min after the feeding, the increased NPQ mechanism at the neighboring area to the feeding spot and at the rest of the leaf area (Figure 4c) not only resulted in decreasing the ETR (Figure 4d), but also reduced the ^1^O_2_ production (Figure 3c).

The decreased Φ*_PSII_* and ETR, at the neighboring area of the feeding spot and at the rest of the leaf area, are attributed to the decreased efficiency of the open PSII reaction centers (F*v′*/F*m′*) (Figure 4b), since there was no change in the number of open PSII reaction centers (q*p*) (Figure 4a). In contrast to our results, 20 min feeding by the leafminer *Tuta absoluta* on potato leaves did not have any effect on F*v′*/F*m′* but decreased the number of open PSII RCs [12]. However, this difference may result from their different feeding styles: *T. absoluta* is a miner, causing internal damage to the leaf, while the Colorado beetle is a chewing herbivore. This is a paradigm that different feeding styles produce differential plant responses.

NPQ can be considered sufficient if the fraction of open PSII RCs can be maintained at a level similar to that in control conditions under any disturbance of plant homeostasis [100,101]. In our study, in agreement with this, the increased NPQ in the neighboring area to the feeding spot and in the rest of the leaf area (Figure 4c) appeared to be sufficient to keep the same portion of PSII RCs open as in controls (Figure 4a). In contrast, after 20 min of feeding by the leafmine *Tuta absoluta* on potato, the increased NPQ could not keep open the same number of PSII RCs as before feeding, suggesting that the NPQ mechanism was not efficient enough [12]. Thus, it can be concluded that potato PSII response mechanism to insect herbivores depends on the insect species.

The enhancement of NPQ after Colorado potato beetle feeding also resulted in decreased ^1^O_2_ generation (Figure 3c). It is now well documented that any disturbance of ROS production at the light reactions of photosynthesis triggers the plant’s protective defense response to environmental perturbations, and it contributes to restoring the “oxidation-reduction” balance [29,37,102,103,104,105,106,107]. The most stable ROS is hydrogen peroxide (H_2_O_2_), which can act as a long-distance signaling molecule mediating plant responses to alterations in homeostasis [108,109,110]. H_2_O_2_ is produced during photosynthesis in the electron transport chain by the electron leakage in PSI that reduces oxygen (O_2_) to superoxide anion (O_2_^•−^), which is rapidly converted to hydrogen peroxide (H_2_O_2_) by the superoxide dismutase (SOD) [29,30,111,112,113]. H_2_O_2_ can travel through leaf veins faster than from cell to cell, and thus it spreads through leaf veins to act as a long-distance molecule triggering the plant defense response during biotic or abiotic stress in plants [12,29,103,114,115].

^1^O_2_ and H_2_O_2_ are the main ROS that initiate various signaling networks when the light reactions of photosynthesis are dysfunctional [116]. ^1^O_2_ is very reactive and can induce but not transduce signaling [117,118]. On the contrary, foliar-produced H_2_O_2_ is less reactive but is a mobile molecule that can diffuse throughout the leaf veins to act as a long-distance messenger [114,116,119]. It seems that the H_2_O_2_ produced at the feeding zone located in the vicinity of the leaf’s midrib (Figure 6b) elicits defense responses to herbivore feeding, by its diffusion through the leaf veins [13,103,120,121]. H_2_O_2_ is the most stable ROS that can mediate plant responses to stress [84]. Since ^1^O_2_ is produced by energy transfer and H_2_O_2_ by electron transport, yet they are produced simultaneously, it appears likely that their signaling pathways can occasionally interfere or antagonize each other [13,29,37,115]. A coordinated ROS generation has been considered the major plant defense response mechanism to herbivores [122].

Plants continuously experience insect herbivory, and insect feeding causes injury and impairs crop production [123,124]. Insect herbivory induces various signals from injured tissues that are perceived in untouched tissues, which subsequently boost their defense [124,125]. Such signals include the phytohormone JA, which is synthesized via the oxygen lipid pathway [126] and regulates plant defense against diverse insect herbivores [123,125,127]. JA biosynthesis proceeds in the vascular bundles [125,128], possibly via hydrogen peroxide signaling. The JA is considered to function as a long-distance signaling molecule traveling through the phloem [125] similar to hydrogen peroxide signaling [13,103,120,121]. The need of plants to protect themselves against herbivores has generated an array of photoprotective mechanisms that permit them to compensate for herbivory [3,129]. The level of plant photoprotective mechanisms also depends on the insect species under examination. Cues derived from different insects differentially influence flavonoid production [130], underlining the importance of the insect species involved in affecting plant-insect interactions.

## 4. Materials and Methods

### 4.1. Plant Material and Growth Conditions

Potato plants (*Solanum tuberosum* L. cv Spunta) were grown in 5 L plastic pots as described before [4]. The growth conditions were 23 ± 2°/19 ± 2 °C (day/night) temperature, 70 ± 5/80 ± 5% day/night relative humidity, and a 14-h photoperiod with photosynthetic photon flux density (PPFD) of 630 ± 10 μmol quanta m^−2^ s^−1^.

### 4.2. Leptinotarsa Decemlineata

Adults of Colorado potato beetle (*L. decemlineata*) (approximately 200 at a 1:1 sex ratio) were collected from a potato field cultivation established at the premises of the Institute of Plant Breeding and Genetic Resources, ELGO-Dimitra (Thermi, Greece). The collected Colorado potato beetle population was maintained at 26 ± 1 °C under a light–dark photoperiod of 16:8 h and 60–65% relative humidity on non-transgenic potato seedlings (*S. tuberosum* L. cv Spunta) in controlled growth chambers. The adults used in the bioassays were starved for 24 h prior to each experiment.

### 4.3. Experimental Design

The 4th terminal leaf of each potato plant was enclosed in the measurement chamber of a fluorometer, and photosynthetic efficiency was measured. Following the first measurement, one randomly selected Colorado potato beetle adult was added and allowed to feed for 20 min without removing the leaflet from the fluorometer’s measurement chamber. After the measurement, the insect was removed, and a new measurement was conducted on the same leaflet immediately after the feeding. Three to four leaves from different potato plants were measured before and after 20 min of Colorado potato beetle feeding.

### 4.4. Chlorophyll Fluorescence Analysis

Chlorophyll fluorescence measurements were performed as described in detail previously [131], using an Imaging-PAM Fluorometer M-Series MINI-Version (Heinz Walz GmbH, Effeltrich, Germany). The potato leaflets were dark-adapted for 20 min before each measurement. The minimum (F*o*) and maximum (F*m*) chlorophyll a fluorescence ιin the dark was recorded, and the variable (F*v*) chlorophyll a fluorescence was calculated from F*v* = F*m *− F*o*. The maximum chlorophyll *a* fluorescence in the light (F*m*’) was acquired with saturating pulses (SPs) every 20 s for 5 min after application of the actinic light (AL) of 636 μmol photons m^−2^ s^−1^, which was selected to match the growing light intensity. The minimum chlorophyll *a* fluorescence in the light (F*o*’) was computed as F*o*’ = F*o*/(F*v*/F*m* + F*o*/F*m*’) [132]. Steady-state photosynthesis (F*s*) was measured after 5 min of illumination time with the AL of 636 μmol photons m^−2^ s^−1^. The remaining chlorophyll fluorescence parameters were estimated using Win V2.41a (Heinz Walz GmbH, Effeltrich, Germany) and are described in detail in Table S1.

### 4.5. Imaging of Hydrogen Peroxide

Hydrogen peroxide (H_2_O_2_) was detected before and after 20 min of Colorado potato beetle feeding on potato leaves. Potato leaflets were incubated in the dark for 30 min with 25 μM 2′,7′-dichlorofluorescein diacetate (DCF-DA, Sigma Aldrich, Chemie GmbH, Schnelldorf, Germany) as described earlier [115,133]. H_2_O_2_-specific fluorescence was observed afterwards with a Zeiss AxioImager Z2 epi-fluorescence microscope equipped with an AxioCam MRc5 digital camera (Carl Zeiss MicroImaging GmbH, Göttingen, Germany) [133].

### 4.6. Statistics

Statistical analysis was performed with R software (version 4.3.1, R Core Team, 2023). A two-way repeated measures ANOVA was conducted for each parameter, followed by post hoc pairwise comparisons using Fisher’s Protected Least Significant Difference (LSD) test. Values were considered significantly different at *p* < 0.05. A linear regression analysis was also performed.

## 5. Conclusions

Colorado potato beetle feeding decreased the maximum efficiency of PSII photochemistry (F*v*/F*m*), suggesting photoinhibition due to reduced efficiency of the oxygen-evolving complex (OEC). The observed reduced amount of absorbed light energy that was directed after feeding to photochemistry (Φ*_PSII_*) was attributed to the reduced efficiency of the open PSII reaction centers (F*v′*/F*m′*), since there was no alteration in the fraction of open PSII reaction centers (q*p*). The ability of potato plants to keep the same fraction of open PSII reaction centers as before feeding suggests an efficient photoprotective mechanism of non-photochemical quenching (NPQ), which was activated to reduce the singlet oxygen (^1^O_2_) formation in potato plants in the neighboring area to the feeding spot and at the rest of the leaf area. Concomitantly, NPQ triggered a slight increase in hydrogen peroxide (H_2_O_2_) production, which can act as a signaling molecule by diffusing through leaf veins to elicit defense responses. A coordinated signaling pathway involving ^1^O_2_ and H_2_O_2_ appears to operate as a biotic stress defense response mechanism in potatoes.

Understanding the molecular mechanisms that initiate adaptive responses in potatoes following Colorado potato beetle feeding is essential for developing new breeding approaches to enhance biotic stress resilience and crop productivity. Our findings highlight the importance of considering both the whole-leaf spatial direct effects of herbivory and the indirect effects on the neighboring area around the feeding spot and the rest of the leaf area.

## Data Availability

All data supporting this study are available within the paper and within its Supplementary Data published online.

## Supplementary Materials

The following supporting information can be downloaded at https://www.mdpi.com/article/10.3390/plants15081159/s1. Table S1: Definitions of the chlorophyll fluorescence parameters used in the experiments.

## Abbreviations

The following abbreviations are used in this manuscript:

^1^O_2_	Singlet oxygen
^3^Chl*	Excited triplet state of chlorophyll
AL	Actinic light
AOIs	Areas of interest
DCF-DA	2′,7′-dichlorofluorescein diacetate
ETR	Electron transport rate
F*m*	Maximum chlorophyll *a* fluorescence in the dark-adapted leaf
*Fm*’	Maximum chlorophyll *a* fluorescence in the light-adapted leaf
*Fo*	Minimum chlorophyll *a* fluorescence in the dark-adapted leaf
*Fo*’	Minimum chlorophyll *a* fluorescence in the light-adapted leaf
*Fs*	Steady-state photosynthesis
*Fv*	Variable chlorophyll *a* fluorescence in the dark-adapted leaf (*Fv* = *Fm* − *Fo*)
F*v’*/F*m’*	Efficiency of the open PSII reaction centers
F*v*/F*m*	Maximum efficiency of PSII photochemistry
F*v*/F*o*	Efficiency of the oxygen-evolving complex on the donor side of PSII
H_2_O_2_	Hydrogen peroxide
JA	Jasmonic acid
NPQ	Non-photochemical quenching (dissipation of excitation energy as heat)
O_2_•^−^	Superoxide anion radical
OEC	Oxygen-evolving complex
PPFD	Photosynthetic photon flux density
PSI	Photosystem I
PSII	Photosystem II
Q_A_	Quinone A
q*p*	Photochemical quenching (fraction of open PSII reaction centers, representing also the redox state of quinone A)
RCs	Reaction centers
ROS	Reactive oxygen species
SA	Salicylic acid
SD	Standard deviation
SOD	Superoxide dismutase
SPs	Saturating pulses
Φ*_NO_*	Quantum yield of non-regulated energy loss in PSII
Φ*_NPQ_*	Quantum yield of regulated non-photochemical energy loss in PSII
Φ*_PSII_*	Effective quantum yield of PSII photochemistry
