# Supplementary material for: Photosystem II Responses at the Whole-Potato-Leaf Level After Colorado Potato Beetle Feeding
Source: Plants (Basel). 2026 Apr 9;15(8):1159. doi: 10.3390/plants15081159 (PMC13119096; doi:10.3390/plants15081159)
Supplement: Supplementary file 1 [file plants-15-01159-s001.zip › plants-4227348-supplementary.pdf]

plants

MDPI

Article

Photosystem II Responses at the Whole Potato Leaf Level After Colorado Potato Beetle Feeding

Ilektra Sperdouli, Stefanos S. Andreadis, Julietta Moustaka, Eleni I. Koutsogeorgiou, Emmanuel Panteris, and Michael Moustakas

Table S1. Definitions of the chlorophyll fluorescence parameters used in the experiments

Parameter	Definition	Calculation
F_o	Minimum chlorophyll *a* fluorescence in the dark-adapted leaf (PSII centers open)	Obtained by applying measuring photon irradiance of 1.2 μmol photons m^{-2} s^{-1}
F_m	Maximum chlorophyll *a* fluorescence in the dark-adapted leaf (PSII centers closed)	Obtained with a saturating pulse (SP) of 6000 μmol photons m^{-2} s^{-1}
F_o'	Minimum chlorophyll *a* fluorescence in the light-adapted leaf	It was computed by the Imaging Win software V2.41a (Heinz Walz GmbH, Effeltrich, Germany) as Fo' =Fo/(Fv/Fm + Fo/Fm')
F_m'	Maximum chlorophyll *a* fluorescence in the light-adapted leaf	Measured with saturating pulses (SPs) every 20 s for 5 min after application of the actinic light (AL) of 636 μmol photons m^{-2} s^{-1}
F_s	Steady-state photosynthesis	Measured after 5 min illumination time before switching off the actinic light (AL) of 636 μmol photons m^{-2} s^{-1}
Φ_{PSII}	Effective quantum yield of PSII photochemistry	(F*m*′ − Fs)/F*m*′
Φ_{NPQ}	Quantum yield of regulated non-photochemical energy loss in PSII	Fs/F*m*′ − Fs/F*m*
Φ_{NO}	Quantum yield of non-regulated energy loss in PSII	Fs/F*m*
Fv/F*m*	Maximum efficiency of PSII photochemistry	(Fm − Fo)/Fm
Fv′/F*m*′	Efficiency of the open PSII reaction centers	(F*m*′ − Fo′)/F*m*′
Fv/Fo	Efficiency of the oxygen evolving complex (OEC) on the donor side of PSII	
ETR	Electron transport rate	Φ_{PSII} × PAR × c × abs, where PAR is the photosynthetically active radiation, c is 0.5, and abs is the total light absorption of the leaf taken as 0.84
q*p*	Photochemical quenching, representing the redox state of quinone A (Q_A), or in other words the fraction of open PSII reaction centers based on the "puddle" model for the photosynthetic unit	(F*m*′ − Fs)/(F*m*′ − Fo′)
NPQ	Non-photochemical quenching reflecting the dissipation of excitation energy as heat	(Fm – Fm′)/Fm′
